# Diagnosis of tuberculous lymphadenopathy: the role of droplet digital PCR

**DOI:** 10.7189/jogh.16.04244

**Published:** 2026-10-09

**Authors:** Yu-Feng Wei, Ching-Yi Chen, Ping-Huai Wang, Yao-Wen Kuo, Chin-Chung Shu, Bo-Shiun Yan

**Affiliations:** 1 School of Medicine for International Students, College of Medicine, I-Shou University, Kaohsiung, Taiwan; 2 Department of Internal Medicine, E-Da Cancer Hospital, Kaohsiung, Taiwan; 3 Division of Chest Medicine, Department of Internal Medicine, E-Da Hospital, Kaohsiung, Taiwan; 4 Department of Internal Medicine, Far Eastern Memorial Hospital, New Taipei City, Taiwan; 5 Department of Integrated Diagnostics & Therapeutics, National Taiwan University Hospital, Taipei, Taiwan; 6 Department of Internal Medicine, College of Medicine, National Taiwan University, Taipei, Taiwan; 7 Department of Internal Medicine, National Taiwan University Hospital, Taipei, Taiwan; 8 Graduate Institute of Clinical Medicine, College of Medicine, National Taiwan University, Taipei, Taiwan; 9 Graduate Institute of Biochemistry and Molecular Biology, College of Medicine, National Taiwan University, Taipei, Taiwan

**Keywords:** droplet digital polymerase chain reaction, *Mycobacterium tuberculosis*, tuberculous lymphadenopathy, formalin-fixed paraffin-embedded tissue

## Abstract

**Background:**

Tuberculous lymphadenopathy (TB-LAP) is the most common form of extrapulmonary TB and remains diagnostically challenging due to its paucibacillary nature. We evaluated the diagnostic performance of droplet digital PCR (ddPCR) for detecting *Mycobacterium tuberculosis* (Mtb) DNA in formalin-fixed paraffin-embedded (FFPE) lymph node specimens.

**Methods:**

We retrospectively analysed 102 lymph node FFPE specimens with microbiologically confirmed or pathology-suspected TB-LAP. We performed ddPCR targeting the IS6110 gene and correlated it with pathological findings and mycobacterial culture results. Diagnostic performance was assessed using receiver operating characteristic analysis.

**Results:**

Pathological findings included granulomatous inflammation (GI) (n = 67), caseating GI (CGI) (n = 22), CGI with acid-fast bacilli (AFB) (n = 7), GI with AFB (n = 1), and atypical findings (n = 5). There was a graded association between ddPCR and pathological severity (*P* for trend = 0.002), with the highest levels in CGI plus AFB and the lowest in non–TB-LAP cases. Mtb DNA concentration (copies/μL) was significantly higher in AFB-positive specimens (mean (x̄) = 22.9, standard deviation (SD) = 61.5) compared with AFB-negative specimens (x̄ = 0.6, SD = 0.8) (*P* = 0.044). Among 52 specimens sent for mycobacterial culture, 9 (17%) were culture-positive, and these specimens had significantly higher ddPCR values (*P* = 0.025). Receiver operating characteristic analysis yielded an area under the curve of 0.715 (95% confidence interval = 0.536–0.894) for AFB positivity. Using a cutoff value of >1.25 copies/μL resulted in 50% sensitivity and 86% specificity. Lowering the cutoff to >0.33 copies/μL increased sensitivity to 88%, but reduced specificity to 51%.

**Conclusions:**

We found that ddPCR quantitatively detects Mtb DNA in lymph node FFPE specimens and correlates with pathological severity and culture positivity. Although the overall diagnostic accuracy was moderate, ddPCR demonstrated distinct sensitivity and specificity profiles at different cutoff values and may serve as a useful adjunctive diagnostic tool when interpreted in conjunction with clinical and histopathological findings in TB-LAP.

Tuberculosis (TB) remains one of the most prevalent infectious diseases worldwide and a leading cause of infection-related mortality [1]. According to the World Health Organization, an estimated 10.7 million new TB cases occurred globally in 2024, resulting in 1.2 million deaths [2]. In Taiwan, 6141 new TB cases were reported in 2024, corresponding to an incidence of 26.2 per 100,000 population [3]. Continued progress toward TB elimination requires further improvements, particularly in early diagnosis, which is a key strategy for improving patient outcomes and reducing transmission [4–6]. However, approximately 15–25% of TB infections involve extrapulmonary sites [7] and are difficult to diagnose because of their paucibacillary nature and atypical clinical presentation [8].

Among them, TB lymphadenopathy (LAP) is one of the most common extrapulmonary manifestations [9], accounting for approximately 25–40% of extrapulmonary TB cases and 10–20% of all TB cases globally [9–11]. A systematic review of tuberculous lymphadenitis in low-incidence countries reported a median total diagnostic and treatment delay of 77.5–122 days [12]. The possible explanations may include the long turnaround time of mycobacterial culture, often requiring several weeks, as well as its only low-to-moderate sensitivity, despite remaining the current diagnostic gold standard [8]. On the other hand, although tissue biopsy for suspected TB-LAP can provide pathological results within days, the procedure is relatively invasive, and typical pathological findings such as granulomatous inflammation (GI) may also be caused by other conditions, including sarcoidosis [13], nontuberculous mycobacterial infection, and fungal infections [14]. Therefore, treatment decisions for TB-LAP based solely on typical pathological findings without microbiological confirmation remain controversial [15,16]. The availability of a rapid microbiological test for LAP tissue to complement pathological findings may substantially improve clinical decision-making; however, studies evaluating such approaches remain limited.

Nucleic acid amplification tests, particularly PCR, have become integral to TB diagnosis by enabling rapid detection of low-level bacterial DNA. However, conventional TB PCR assays are primarily indicated for respiratory samples, and their performance in non-respiratory or pathological specimens remains suboptimal. In formalin-fixed pathological specimens, DNA fragmentation further reduces sensitivity, and real-time PCR achieves only 40–60% sensitivity in pathological specimens [17,18] which remains insufficient for clinical needs. Droplet digital PCR (ddPCR) represents a newer molecular technology that partitions DNA into thousands of microdroplets, allowing absolute quantification and improved detection of low-abundance DNA [19]. Compared with conventional real-time PCR, ddPCR may offer enhanced analytical sensitivity and improved quantitative precision. While ddPCR has demonstrated promising performance in sputum and blood specimens [20–23], its application in pathological tissue remains challenging. To date, only two small-scale studies (60–80 specimens) have evaluated ddPCR in pathological samples, reporting sensitivities of 57.8% [17] and 98.5% [24] with considerable variability. Larger and more rigorous validation studies are therefore warranted.

To address this gap, we aim to evaluate ddPCR for *Mycobacterium tuberculosis* (Mtb) DNA detection in pathological LAP specimens from patients with suspected TB-LAP. By assessing the diagnostic accuracy and adjunctive value of ddPCR, we seek to reduce diagnostic uncertainty and improve clinical decision-making in TB-LAP.

## METHODS

### Participant enrolment

We conducted a retrospective cohort study from December 2021 to March 2025 in two medical centres (National Taiwan University Hospital and E-Da Memorial Hospital) in Taiwan. We included patients aged ≥20 years with microbiology confirmed diagnosis of TB-LAP or lymph node tissue showing GI, dividing them into two groups based on clinical diagnosis: TB-LAP and non-TB-LAP. The TB-LAP group was defined by microbiologically (culture) confirmed Mtb infection or clinical diagnosis by pathological findings plus treatment response to anti-TB therapy [25,26]. Mycobacterial culture was performed according to routine clinical practice. We excluded patients with AIDS and no residual LAP tissue in the hospital.

### Sample collection

We used LAP residual formalin-fixed paraffin-embedded (FFPE) tissue stored in the Department of Pathology in the study hospitals. Our fixation time was routinely around 6–72 hours, and storage conditions were at room temperature of 20–25°C. The mean (x̄) block age was 3.78 years (standard deviation (SD) = 0.66). We used the Qiagen Kit (QIAGEN, USA), which uses ten 10 μm-thick FFPE sections for DNA extraction, following the manufacturer’s instructions. All samples were stored at −80°C immediately until analysis.

### ddPCR

We examined the presence of Mtb bacilli in the DNA of FFPE samples by ddPCR targeting the IS6110 gene of Mtb. We used the forward primer (GACCTACTACGACCACATCAACCGGGAGCCC) and reverse primer (GTTCAGGGTTAGCCACACTT) for IS6110 amplification. Using a T100 Thermal Cycler (Bio-Rad, USA), a ddPCR-based detection method was performed using 40 ng cfDNA with QX200 ddPCR EvaGreen supermix (Bio-Rad, USA) and Droplet Generation Oil for EvaGreen (Bio-Rad, USA) according to the protocol from the manufacturer. The process was started at 95°C for 5 minutes, followed by 40 cycles of 95°C for 30 seconds and then 58°C for 1 minute, and finally at 4°C for 6 minutes, 90°C for 5 minutes, and at 12°C. The results were read using a QX200 Droplet Reader (Bio-Rad, USA).

### Statistical analysis

Data were expressed as x̄ (SD) or n (%). We compared continuous variables using the Mann-Whitney U test and categorical variables using Fisher exact test. We conducted a receiver operating characteristic curve analysis to determine the diagnostic performance and optimal cut-off values for Mtb DNA in LAP FFPE tissue. Generalised estimating equation (GEE) analyses were performed for multiple specimens obtained from the same patient, and multivariable logistic regression was also performed. We conducted additional GEE analyses evaluating the association between pathological categories (five groups) and continuous ddPCR values, including calculation of the overall joint *P*-value.

We used SPSS, version 19 (SPSS Inc., Chicago, Illinois, USA) for all analyses. We used Python, version 3.11 (Python Software Foundation, Beaverton, Oregon, USA) for additional GEE analyses. A *P*-value of <0.05 was considered statistically significant.

## RESULTS

### Enrolled participant

During the study period, we enrolled 102 lymph node FFPE specimens from 47 patients with suspected tuberculous lymphadenitis. We obtained multiple specimens from the same individuals as part of routine clinical practice. Therefore, the analyses were primarily performed at the specimen level. We extracted DNA from these specimens and analysed it using ddPCR. Among them, 56 (55%) were tissue from mediastinal LAP, 38 (37%) from neck LAP or mass, 7 (7%) from intra-abdominal LAP or tissue, and 1 (0.9%) from inguinal LAP.

### Pathology findings and mycobacterial culture

The pathological classifications included 67 GI cases, 22 caseating GI (CGI) cases, 7 CGI cases with acid-fast bacilli (AFB), 1 GI case with AFB, and 5 atypical findings (4 anthracosis-related GI and 1 suppurative inflammation). Among them, 38 (37%) specimens were from those receiving anti-TB treatment and 64 (63%) from those not receiving treatment. From 52 specimens sent for mycobacterial culture, positive Mtb were isolated in 9 (17%) specimens (including 5 CGI, 3 CGI plus AFB, and 1 GI). One case isolated nontuberculous mycobacteria in a specimen with GI plus AFB pathological findings. Mycobacterial culture was positive in 2.9% (n/N = 1/34) of the pathological GI findings, whereas it was positive in 62.5% (n/N = 5/8) of the CGI findings. Among the 8 AFB-positive specimens, culture results were available for 6 specimens, of which 3 (50%) showed Mtb.

### Demographic characteristics

The overall x̄ age was 54.9 years (SD = 19.3), and the sample was female-predominant (n = 64, 63%) (**Table 1**). Patients who received treatment (x̄ = 49.3, SD = 19.0) were significantly younger than those without treatment (x̄ = 58.1, SD = 18.9) (*P* = 0.025). Age did not significantly differ by AFB status or culture results. There were no significant differences in sex distribution or body mass index across the treatment, AFB, and culture groups. Smoking status differed across treatment and culture groups, but not by AFB status. Notably, patients without treatment were more likely to be never-smokers (89%) compared to those with treatment (74%) (*P* = 0.035), and culture-positive patients had a higher proportion of current smokers (22%) compared with culture-negative patients (0%) (*P* = 0.004). The prevalence of malignancy, diabetes mellitus, long-term dialysis, and autoimmune disease did not significantly differ across treatment, AFB, or culture subgroups.

**Table 1:** Clinical characteristics of enrolled participants by TB treatment, AFB in the pathology findings, and Mtb culture in the tissue*

		Treatment status			AFB status			Mtb culture status†		
	**All (n = 102)**	**Treatment (n = 38)**	**No treatment (n = 64)**	***P*-value**	**Positive (n = 8)**	**Negative (n = 94)**	***P*-value**	**Positive (n = 9)**	**Negative (n = 43)**	***P*-value**
**Age (in years), x̄ (SD)**	54.9 (19.3)	49.3 (19.0)	58.1 (18.9)	0.025	54.2 (24.9)	54.9 (18.9)	0.645	51.5 (20.1)	53.4 (12.9)	0.797
**Male sex**	38 (37)	16 (42)	22 (34)	0.133	5 (63)	35 (37)	0.257	6 (67)	15 (35)	0.215
**BMI (in kg/m^2^), x̄ (SD)**	23.3 (5.4)	22.6 (4.0)	23.7 (6.1)	0.329	22.6 (4.7)	23.3 (5.5)	0.896	25.3 (4.0)	23.9 (6.0)	0.982
**Smoking**				0.035			0.420			0.004
Current	3 (3)	3 (8)	0 (0)		0 (0)	14 (15)		2 (22)	0 (0)	
Quit	14 (14)	7 (18)	7 (11)		0 (0)	3 (3)		0 (0)	7 (16)	
Never	85 (83)	28 (74)	57 (89)		8 (100)	77 (82)		7 (78)	36 (84)	
**Underlying co-morbidity**										
Malignancy	2 (2)	2 (5)	0 (0)	0.136	0 (0)	2 (2)	0.849	0 (0)	0 (0)	N/A
Diabetes mellitus	21 (21)	7 (18)	14 (22)	0.440	2 (25)	19 (20)	0.520	5 (56)	13 (30)	0.143
Long-term dialysis	2 (2)	2 (5)	0 (0)	0.136	0 (0)	2 (2)	0.849	0 (0)	0 (0)	N/A
Autoimmune disease	7 (7)	1 (3)	6 (9)	0.188	1 (13)	6 (6)	0.446	0 (0)	1 (2)	0.827
**Location**				<0.001			<0.001			<0.001
Neck	38 (37)	29 (76)	9 (14)		6 (75)	32 (34)		8 (89)	6 (14)	
Mediastinum	56 (55)	8 (21)	48 (75)		0 (0)	56 (60)		1 (11)	36 (84)	
Intra-abdominal	7 (7)	0 (0)	7 (11)		1 (13)	6 (6)		0 (0)	1 (2)	
Inguinal	1 (1)	1 (3)	0 (0)		1 (13)	0 (0)		0 (0)	0 (0)	

AFB – acid-fast bacilli, BMI – body mass index, Mtb – Mycobacterium tuberculosis, SD – standard deviation, TB – tuberculosis, x̄ – mean

*Values are presented as n (%) unless specified otherwise.

†Only 52 specimens were sent for mycobacterial culture.

### Lesion location

Lesion location showed significant differences across all three subgroup comparisons (all *P* < 0.001). Among treated patients, cervical (neck) involvement was the most common (76%), whereas mediastinal involvement predominated in untreated patients (60%). Similarly, AFB-positive specimens were primarily located in the neck (75%), while AFB-negative cases were more commonly mediastinal (60%). Culture-positive specimens were predominantly cervical (83%), whereas culture-negative cases were more often mediastinal (74%).

### Conventional PCR results

Conventional PCR was performed in 19 specimens, including 3 specimens with CGI plus AFB and 16 specimens with GI. The average Mtb DNA level was higher among specimens with CGI plus AFB (x̄ = 20.3, SD = 58.0) than in those with GI (x̄ = 0.7, SD = 0.8). All conventional PCR results were negative, including four culture-positive specimens.

### ddPCR results

By using ddPCR to check Mtb DNA in the FFPE pathology specimens (**Table 2**), we found that the average number of positive droplets was higher in treated (x̄ = 71.3, SD = 365.8) than in untreated patients (x̄ = 8.8, SD = 10.4), but the difference was not statistically significant (*P* = 0.299). We also found that AFB-positive specimens had markedly higher droplet counts (x̄ = 297.5, SD = 795.2) than AFB-negative cases (x̄ = 9.5, SD = 11.8), with a trend toward significance (*P* = 0.063). Similarly, ddPCR concentration (copies/μL) was significantly higher in AFB-positive specimens (x̄ = 22.9, SD = 61.5) than in AFB-negative cases (x̄ = 0.6, SD = 0.8) (*P* = 0.044). Across different pathological categories, ddPCR results were the highest in specimens with CGI plus AFB positivity, followed by CGI without AFB and GI, and were lowest in cases ultimately excluded as TB-LAP (both *P* for trend = 0.002) (**Figure 1**).

**Table 2:** ddPCR results by TB treatment, AFB in the pathology findings, and Mtb culture in the tissue*

		Treatment status			AFB status			Mtb culture status		
	**All (n = 102)**	**Treatment (n = 38)**	**No treatment (n = 64)**	***P*-value**	**Positive (n = 8)**	**Negative (n = 94)**	***P*-value**	**Positive (n = 9)**	**Negative (n = 43)**	***P*-value**
**ddPCR quantification of Mtb DNA**										
Positive droplets	32.1 (223.6)	71.3 (365.8)	8.8 (10.4)	0.299	297.5 (795.2)	9.5 (11.8)	0.063	264.3 (750.4)	11.0 (13.2)	0.026
Concentrations (copies/μL)†	2.4 (17.3)	5.4 (28.3)	0.5 (0.7)	0.298	22.9 (61.5)	0.6 (0.8)	0.044	20.3 (58.0)	0.7 (0.8)	0.025

AFB – acid-fast bacilli, BMI – body mass index, ddPCR – droplet digital PCR, Mtb – Mycobacterium tuberculosis, SD – standard deviation, TB – tuberculosis, x̄ – mean

*Values are presented as mean (standard deviation).

†The data have been corrected by negative control.

**Figure 1:**
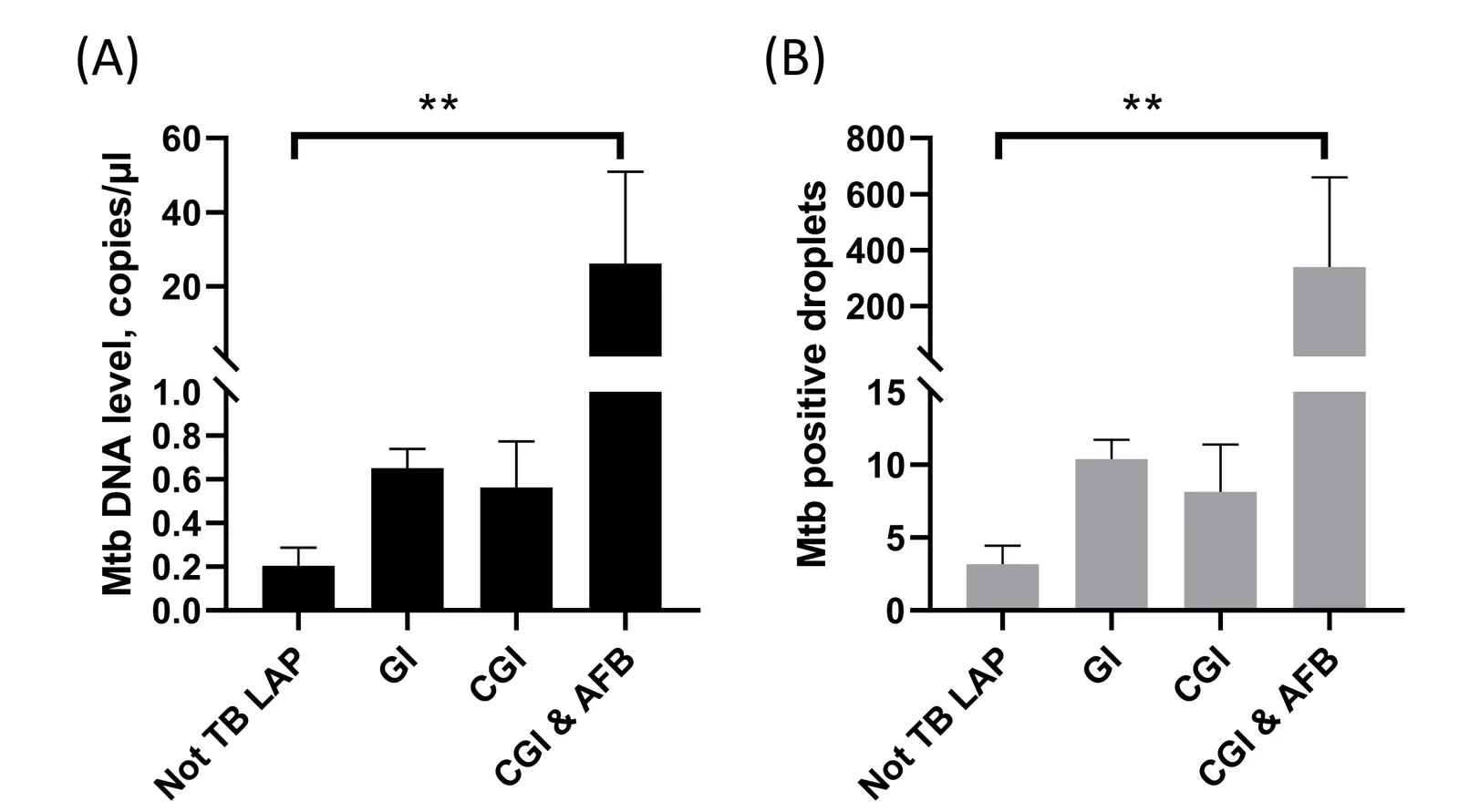
Receiver operating characteristic curves of Mtb DNA concentration and positive droplet counts for detecting pathological AFB positivity. AUC – area under the curve, ddPCR – droplet digital PCR, Mtb – Mycobacterium tuberculosis, ROC – receiver operating characteristic, TB – tuberculosis.

Among specimens with available Mtb culture results, ddPCR values were significantly higher in culture-positive (x̄ = 264.3, SD = 750.4) than in culture-negative (x̄ = 11.0, SD = 13.2) samples in terms of positive droplet counts (*P* = 0.026). In terms of DNA concentration (copies/μL), the values were also higher in culture-positive (x̄ = 20.3, SD = 58.0) compared to culture-negative (x̄ = 0.7, SD = 0.8) samples (*P* = 0.025).

For sensitivity analysis using the first specimen per patient, ddPCR could still discriminate Mtb DNA levels across different pathology severities (*P* for trend = 0.035), positive AFB *vs.* no AFB (*P* = 0.003), and positive *vs.* negative culture (*P* = 0.073). In addition, the results analysed by the GEE model were still significant between different pathological findings and ddPCR results (*P* < 0.0001).

Using pathological AFB positivity as the reference standard, ddPCR achieved an area under the receiver operating characteristic curve of 0.715 (95% confidence interval (CI) = 0.536–0.894, *P* = 0.044) by Mtb DNA concentration and 0.698 (95% CI = 0.518–0.878, *P* = 0.064) by positive droplet count (**Figure 2**). Based on the Youden index, a cutoff value of >1.25 copies/μL yielded a sensitivity of 50% and specificity of 86% for detecting pathological AFB positivity. In multivariable GEE logistic regression analysis, Mtb DNA concentrations >1.25 copies/μL measured by ddPCR remained significantly associated with pathological AFB positivity in FFPE LAP samples (adjusted odds ratio = 4.50, 95% CI = 1.15–17.58, *P* = 0.030) after adjustment for age, sex, and lesion location.

**Figure 2:**
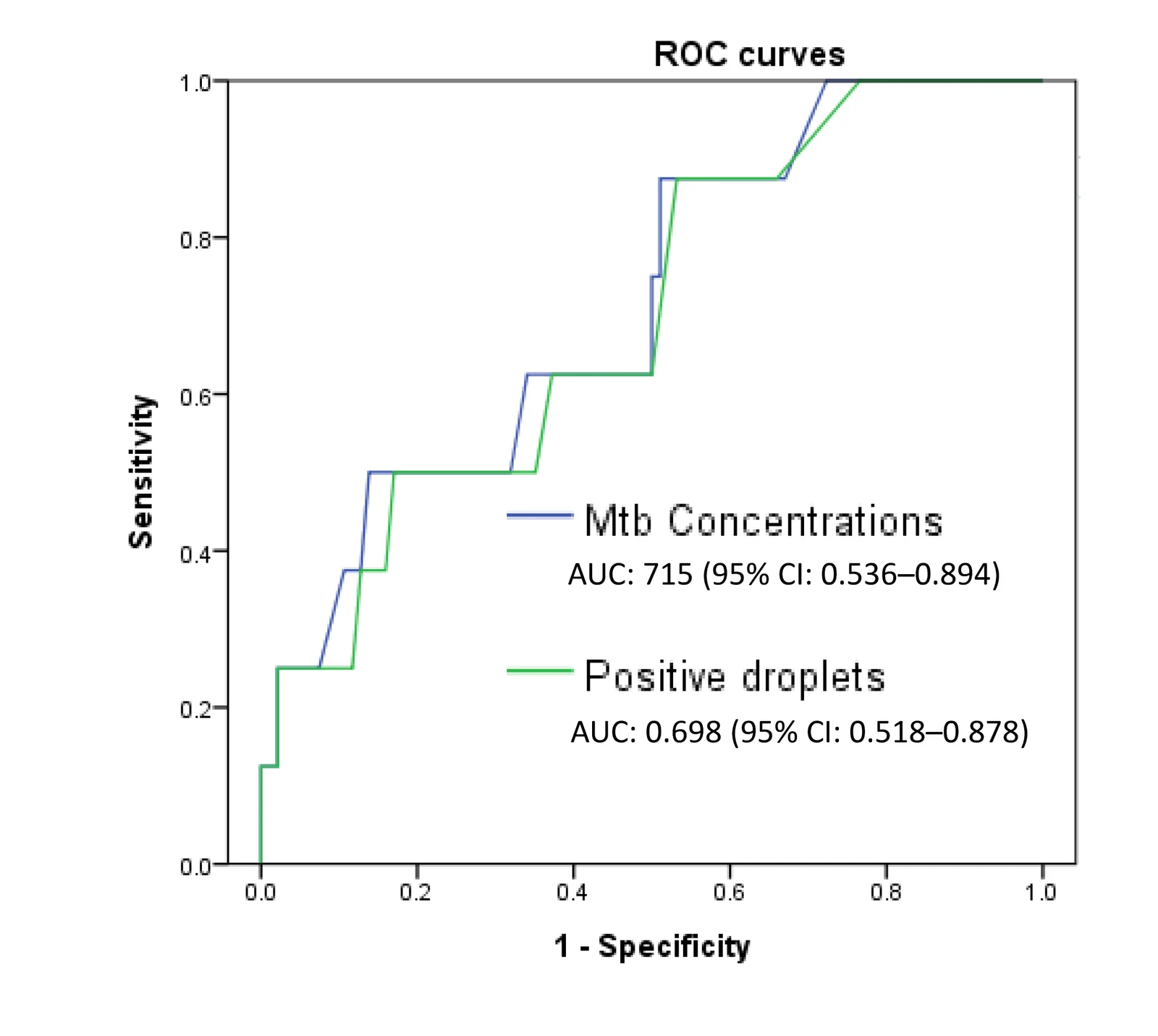
Mtb DNA concentrations and positive droplet counts measured by droplet digital PCR across histopathological categories. Panel A. Mtb DNA concentration (copies/μl). Panel B. Mtb-positive droplet counts. AFB – acid-fast bacilli, CGI – caseating granulomatous inflammation, GI – granulomatous inflammation, LAP – lymphadenopathy, Mtb – Mycobacterium tuberculosis, TB – tuberculosis.

## DISCUSSION

In this multicentre study, we evaluated ddPCR for detecting Mtb DNA in lymph node FFPE specimens from patients with suspected TB-LAP. Our findings show that ddPCR provides quantitative molecular data that correlate with histopathological severity and culture positivity, supporting its potential role as an adjunctive diagnostic tool in extrapulmonary TB, particularly when pathological features are suggestive and microbiology is inconclusive. By providing additional microbiological support in smear- or culture-negative cases, ddPCR may assist clinical decision-making regarding empirical anti-TB therapy and improve diagnostic confidence.

Globally, TB-LAP is the most common form of extrapulmonary TB, accounting for approximately 25–40% of extrapulmonary TB cases [9–11]. However, diagnosis remains challenging due to the paucibacillary nature of the disease, low smear sensitivity, and variable culture yield [8]. We observed a biologically coherent gradient between ddPCR output and histopathological categories. Positive droplet counts and DNA concentrations were significantly higher in AFB-positive and caseating lesions. This aligns with established understanding that bacillary burden is greatest in necrotising granulomas and lowest in non-caseating inflammation [27].

Conventional PCR in FFPE tissue has shown variable and suboptimal sensitivity, partly because of DNA fragmentation and low template abundance [17,18]. In our cohort, all conventional PCR results were negative. In contrast, ddPCR partitions samples into thousands of droplets, enabling absolute quantification and improved detection of low-copy targets [28,29]. Previous studies evaluating ddPCR in FFPE samples mainly included confirmed TB-positive specimens or heterogeneous TB samples, while the number of lymphadenitis/TB-LAP cases was relatively small [17,24]. In addition, evidence specifically focusing on clinically suspected TB-LAP remains limited. Therefore, we provide a novel perspective by evaluating the clinical usefulness of ddPCR in patients with suspected TB-LAP. In our cohort, using pathological AFB as reference, ddPCR achieved an area under the curve of 0.715. A cutoff of >1.25 copies/μL demonstrated relatively high specificity (86%) but limited sensitivity (50%); therefore, it may be more useful in supporting a high clinical suspicion of TB-LAP diagnosis. In contrast, lowering the cutoff to >0.33 copies/μL increased sensitivity to 88% at the expense of reduced specificity (51%), suggesting potential utility as a screening approach.

Culture remains the microbiological gold standard but is limited by delayed turnaround and modest sensitivity in extrapulmonary specimens [30]. In our study, culture positivity was found in only 17% of specimens and was significantly associated with higher ddPCR droplet counts and Mtb DNA concentrations, consistent with prior observations that molecular quantification reflects bacterial burden [17]. Notably, all conventional PCR results were negative in our cohort, including some culture-positive cases, consistent with prior reports of suboptimal PCR performance in FFPE tissue [27]. However, the mycobacterial culture was performed according to routine clinical practice and might have introduced selection bias, potentially enriching the culture-positive subgroup with more advanced disease and inflating the apparent correlation between ddPCR and culture positivity. Therefore, the results warrant further validation.

Given that extrapulmonary TB diagnosis often relies on invasive sampling and histology due to low microbiological yield [30], a quantitative molecular adjunct may strengthen clinical confidence, particularly in moderate TB-burden settings such as Taiwan [3]. Although ddPCR cannot distinguish viable from nonviable bacilli, its quantitative output may provide biologically relevant information that complements histopathology.

This study has several limitations. First, the sample size remains moderate and AFB-positive specimens were limited. Second, culture results were unavailable for 50 specimens, while conventional Mtb PCR results were unavailable for 83 specimens. This is because these tests were performed according to routine clinical practice rather than a predefined study protocol. We therefore used pathological AFB positivity as the reference standard for ddPCR performance evaluation, primarily because only a limited number of specimens were sent for mycobacterial culture. This limitation may have reduced the comprehensiveness of the diagnostic accuracy assessment against the microbiological gold standard. Third, the diagnosis of TB-LAP was defined by microbiological confirmation and clinical diagnosis, which may be confounded by clinician confirmation bias. Fourth, multiple specimens were obtained from some patients, which may have introduced intra-individual clustering and potentially violated the assumption of statistical independence. However, we validated the data using one specimen per patient and a GEE model. Furthermore, ddPCR detects DNA fragments and cannot assess organism viability. Additionally, although all FFPE specimens were processed under routine clinical conditions at our institution, we cannot exclude residual variability related to FFPE conditions. Finally, IS6110 copy number variation among Mtb strains may affect quantitative interpretation [31].

## CONCLUSIONS

Our results show that ddPCR enables quantitative detection of Mtb DNA in lymph node FFPE specimens and correlates with pathological severity and culture positivity. Although it does not replace culture, ddPCR may serve as a potential adjunctive diagnostic tool in TB-LAP, supporting clinical decision-making regarding anti-TB therapy and improving early diagnosis.

## Data Availability

**Data availability:** The data from this study should be shared after obtaining permission from the Research Ethics Committee of the study hospitals.
